# Nutritional and health status of Afghan refugee women living in Punjab: A cross‐sectional study

**DOI:** 10.1002/fsn3.3267

**Published:** 2023-03-28

**Authors:** Maleeha Fatima, Farhana Nosheen, Muhammad Afzaal, Fakhar Islam, Rabia Noreen, Ali Imran, Yuosra Amer Ali

**Affiliations:** ^1^ Department of Home Economics Government College University Faisalabad Pakistan; ^2^ Department of Food Sciences Government College University Faisalabad Pakistan; ^3^ Department of Food Sciences, College of Agriculture and Forestry University of Mosul Mosul Iraq

**Keywords:** Afghan refugees, body mass index, malnutrition, nutritional status, prevalence

## Abstract

Afghan refugees are the world's densely populated community, with 2.6 million registered Afghan refugees living globally, of whom about 2.2 million are in Iran and Pakistan. Pakistan is a densely populated country, and due to its low socioeconomic status, food insecurity, unhygienic conditions, and inadequate access to health care, the Afghan refugees have high chances of being malnourished; the chances of death for these Afghans are 25 times more every year from undernourishment and poverty than those from violence. This study aimed to assess anthropometric and biochemical analyses, their health complications, and the socioeconomic status of Afghan refugee women living in Islamabad Punjab as women are the most vulnerable and highly malnourished group in any community. This cross‐sectional study involved 150 Afghan women aged 15–30 years; they were assessed for their nutritional status using Anthropometric, Biochemical, Clinical and Diet (ABCD). The results indicate the prevalence of underweight, normal weight, and overweight at 74.7%, 16.7%, and 8.7%, respectively. Majority of the women have extremely low hemoglobin (Hb) level, which indicates iron deficiency as well as low body mass index for their age. As the results indicate that there are high chances of severe malnutrition among this most vulnerable segment of the population, this situation must be addressed immediately; the main aim of this study is to highlight the current situation of these Afghan refugees in Pakistan. More research is needed to determine how normal body weight women with low hemoglobin levels are compared to women with ideal body mass index ratios.

## Graphical abstract

A majority of the women have extremely low Hb level, which indicates iron deficiency as well as low body mass index with regard to their age. The results indicate that there are high chances of severe malnutrition among this most vulnerable segment of the population, and this situation must be addressed immediately.

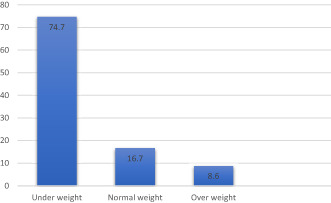

## 1 INTRODUCTION

A balanced and healthy diet plays a vital role in promoting healthy pregnancy, providing healthy growth and development, providing a strong immune system, and lowering the risk of chronic and other noncommunicable diseases (Yamaguchi et al., 2022). Eating low quantities of healthy foods causes challenges and imbalances with social determinants of health (Dong et al., 2022). Nutrition provides an appropriate food pattern to prevent infection through optimal immune response (Hamidianshirazi et al., 2022) and helps in promoting diverse gut microbiota that supports the immune system (Calder, 2021). Proper nutrition and healthy eating habits are essential for survival, physical development, mental health, performance, and overall health (Khan, Khanlou, et al., 2018), whereas household food insecurity leads to poor physical and mental health, frequent hospitalizations, and premature deaths of infants (Hutchinson & Tarasuk, 2022). Maternal nutrition involves nutrition during antenatal or postnatal stages and also the time before conception and even starting from adolescence when early childbearing is normal (Kim et al., 2020). Adolescent population mostly experiences nutritional challenges, which strongly impacts their growth and development (Kumar, 2015). Underweight and nutritional inadequacies are high among teenage girls of reproductive age in low‐ and middle‐income countries (Caulfield & Elliot, 2015). Anemia, iron deficiency, is a global public health problem and is prevalent in both developing and developed countries (Harni, 2022). It affects 2 billion people globally, including 30%–50% of pregnant women (Peña‐Rosas et al., 2015); also pregnant women in developing countries are affected by anemia (Gupta et al., 2022). The World Health Organization also estimated that 50% of pregnant women globally are anemic and that anemia is more common among pregnant women in South Asia (Parks et al., 2019). Anemia affects almost one‐third of the global population, 42% of children and 40% of pregnant women (Kakkar & Aundhakar, 2022). The prevalence of iron deficiency is higher in menstruating women than in men (Gybel‐Brask et al., 2018). Menstruation in women is considered the main factor for the decrease in iron reserves in the body (Chai et al., 2021). Poverty, famine, hunger, war, interethnic struggles, and class oppression cause millions of people to be displaced from their home countries and live as refugees in host countries (Malik et al., 2019). Refugees fear for a number of reasons like race, religion, membership of a specific group, or political opinion (Pakravan‐Charvadeh et al., 2021). War affects areas where people reside, and Afghanistan is one of the major countries that experience war for almost three decades (Alemi et al., 2014). Refugees face minority status problem, socioeconomic stress, lack of social support, and psychological discomfort (Amstutz et al., 2020), and are affected by infectious and noncommunicable illnesses (Taherifard et al., 2021).

Women serve as gatekeepers of nations and also as mentors deciding the daily intakes of family (Silvestrin et al., 2013). Women's education is required for improving child nutrition in low‐ and middle‐income countries (Bras & Mandemakers, 2022). The UNICEF reported that malnutrition affects all sections of the community, but mainly infants and adolescents are more affected (Steele et al., 2016). Malnutrition is the unhealthy condition of body due to imbalance of energy or nutrients (Aylward et al., 2021), and malnourished girls will eventually be malnourished mothers in future (Ahmed & Haboubi, 2010); also they have preterm births because of their poor nutritional status (Badshah et al., 2008). Refugees face a high risk of malnutrition due to poor socioeconomic status, food insecurity, and insufficient health‐care facility (Saeedullah et al., 2021). Malnutrition is directly or indirectly responsible for maternal mortality and is particularly high in Afghanistan (Kim et al., 2020). Afghans are staunch Muslims (Malik et al., 2019), and Afghan women are less educated than men, marry at a young age, and have many babies until a boy is born (Smith, 2009). Afghans are affected by undernourishment and poverty, causing a high death rate among them (Akseer et al., 2018). Marriage at a young age causes adverse effects such as pregnancy complications, anemia, preterm delivery, postpartum infections, low‐birth‐weight (LBW) infants, and children with congenital abnormalities (Palupi & Rizki, 2020). Both maternal height and early‐pregnancy body mass index (BMI) are considered main indicators in accessing birth weight of newborn babies (Spada et al., 2018), and LBW babies are born mostly in developing countries more than in developed countries due to poor maternal health and nutrition (Bansal et al., 2019). Marriage age is the most important factor that impacts reproductive health, fertility, mortality, and health outcomes (Čvorović, 2022). The World Bank has placed Pakistan among the low‐ and middle‐income countries (World Bank Country and Lending Groups, 2021) with an annual household income per capita as just $587 (CEIC Data, 2021). Huge numbers of Afghans seek refuge in the neighboring country of Pakistan (Kassam & Nanji, 2006). The latest reports of the United Nations High Commission for Refugees state that although Pakistan is facing financial and political instability, it is ranked as the third country in the world to provide shelter for a large number of refugees (UNHCR, 2020). The main aim of this study is to assess anthropometric as well as biochemical analysis of Afghan refugees, their health‐related complications, and their socioeconomic status.

## 2 MATERIALS AND METHODS

This study was conducted in Islamabad under the supervision of the Department of Home Economics, Government College University, Faisalabad.

### 2.1 Study design

The study was approved by the Institutional Animal Care and Ethics Committee (ERC 5214). The likelihood and degree of discomfort to human/animal subjects expected during this study were not greater than those usually faced in daily life or during the performance of routine physical examinations or tests. Furthermore, in this study, no genetically modified organism was used, and there was neither any hazard to the environment nor any chance of COVID‐19 dissemination. We followed the tenets of the Declaration of Helsinki and Stockholm Convention. Moreover, the human subjects were enrolled after obtaining consent after specific inclusion and exclusion criteria. Furthermore, before the research all the subjects were again asked for their final willingness. It was a cross‐sectional study in which women were chosen from different age groups and studied at one specific point simultaneously. The cross‐sectional study included a nutritional questionnaire for assessment of their nutritional health status.

### 2.2 Setting

Afghan refugees live almost in every city of Pakistan, but the majority are in Peshawar, Rawalpindi, and Islamabad due to the proximity of these cities to the Pakistan–Afghanistan border. The research was conducted in Islamabad. Afghan refugees were allowed to live in different places as directed by the government of Pakistan. The research area was located in sector H‐12 behind the boundary of NUST University main campus, where about 2000–3000 Afghan refugees lived in muddy houses. The characteristics of respondents are presented in Table 1.

**TABLE 1:** Characteristics of respondents

Age group	Sex	Year of arrival in Pakistan	Languages spoken
15–30 years	Female	2000–2020	Afghan Pashto Dari

### 2.3 Duration of study and sample size

The maximum duration of the research was 6 months. One hundred fifty randomly selected women were included in our research, and the nutritional health status of 50 of the 150 participants was assessed using both anthropometric assessment and Hb meter. The remaining 100 participants’ nutritional health status was assessed using anthropometric assessment. As samples were selected through convenient sampling, only those participants who consented to participate were included in this research study. In this type of sampling, we selected only those who were easily available, were easily approachable, and agreed to participate in the research.

### 2.4 Data collection

Data were collected in the form of a questionnaire. Data on the nutritional health status were collected from women aged 15–30 years. A Hb apparatus was used to identify iron deficiency in the sample, and using these measurements, we were able to identify the current malnutrition ratio in Afghan refugees.

### 2.5 Sociodemographic data collection

General information such as name, age, gender, and father/husband name as well as socioeconomic status such as marital status, monthly income, education level, and family size was collected because all these four factors affected their socioeconomic status directly and indirectly, and this information was collected through detailed discussion with the participants.

### 2.6 Anthropometric measurements

For anthropometric measures, first, height and weight of the participants were measured using the following standard methods. For height measurement, participants were asked to remove footwear and socks and stand stable on a wall in a horizontal position. Height was recorded, and weight was recorded in kilograms using a weighing machine. BMI was then calculated by the fraction of weight to the squared height (kg/m^2^) (Table 2).

**TABLE 2:** Different BMI categories

BMI categories
Underweight: <18.5 kg/m^2^
Normal weight: 18.5–24.9 kg/m^2^
Overweight: 25–29.9 kg/m^2^
Obese: ≥30 kg/m^2^
*Source:* Alshahrani et al. (2016).

Abbreviation: BMI, body mass index.

### 2.7 Biochemical assessment

Biochemical assessment was performed by hemoglobin‐level measurements using a Hb meter. Using the hemoglobin level, RBC (red blood cell) count in the blood was found, and RBC count is the direct indicator of anemic condition. Iron deficiency cannot be identified easily based only on hemoglobin concentration. Not all cases of anemia are caused by iron deficiency. But hemoglobin concentration shows the degree of iron deficiency. The Hb meter showed different readings when strips were used to prick blood from any of the fingers. It was a quick, simple, inexpensive, and reliable indicator for checking the presence and severity of anemia. Before the blood sample was obtained, participants washed their hands, and the finger from which blood was collected was rubbed with clean cotton to avoid contamination.

### 2.8 Data analysis

Many software programs like SPSS (Statistical Package for Social Sciences), Excel, and Minitab are used, but we used SPSS for data entry; using SPSS, graphs, mean, median, mode, standard deviation, and other required results were obtained. The numeric variables like height, weight, sleeping hours, age, and BMI were presented in the form of mean and standard deviation. Categorical variables like lifestyle habits, presence of any disease, occupation, food preferences, and food allergies were presented in the form of frequencies and percentages. Based on the form of data, different suitable tests were applied for obtaining results.

## 3 RESULTS AND DISCUSSION

Malnutrition is one of the main causes of different health complications in Afghan women due to inadequate dietary intake. The World Bank reported that Afghanistan that is on top for having percentage of about 41% in South Asian countries (Chakrabarty, 2022).

### 3.1 Sociodemographic status

Some important factors for assessing nutritional status are (1) education level, (2) family size, (3) monthly income, and (4) marital status. Afghan refugees are one of the population groups that have a low socioeconomic status. Education is the keystone of state development, and mostly developing countries have a much higher fertility rate as well as a lower education level than developed countries (Shen, 2017). The results of this study revealed that only 16.67% of participants are literate and the remaining 93.33% participants are illiterate. Knowledge, attitude, and practice of family planning are less in developing countries due to reasons such as lack of education, resources, and poverty (Sultan, 2018). The family size of these Afghan refugees was large than the other communities of Punjab. The majority of these participants had a family size of 11–12 members, which is very large with regard to their living standards and their income level; 76% of the participants had an income of ₹11,000–15,000, which is very low and insufficient for feeding a large family, and 9.3% of participants had an income of ₹16,000–20,000. The lowest income level among these participants, 14.7%, was ₹5000–10,000. The majority of participants were unmarried; 54% belonged to the age group 15–20 years, and 46% of participants were married. The association between education level and BMI using *χ* ^2^ test is presented Table 3.

**TABLE 3:** Association between education level and BMI using *χ* ^2^ test

Education level	Under weight	Normal weight	Over weight	Total
Literate	4	5	1	10
Percentage within their education level	40.0%	50.0%	10.0%	100%
Illiterate	108	20	12	140
Percentage within their education level	77.1%	14.3%	8.6%	100%
Total	112	25	13	150
Percentage within their education level	74.7%	16.7%	8.7%	100%

Abbreviation: BMI, body mass index.

**FIGURE 1:**
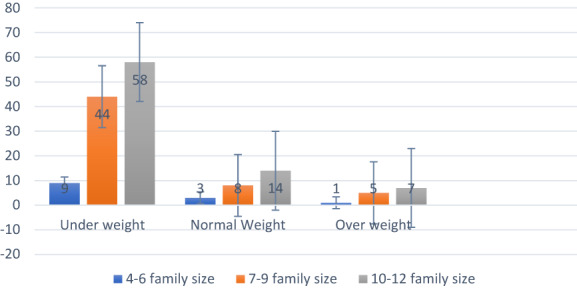
Bar graph showing family size and frequencies based on their BMI (body mass index)

*p‐*Value is .012 with a Pearson's *χ* ^2^ value of 8.889, which shows a significant relation between education and BMI. This is less than 0.05, meaning education strongly affected BMI ratio. Figure 1 shows a bar graph of family size and frequencies based on their BMI.

Table 4 shows that the mean of the education level of participants is 1.93 with a standard deviation of 0.250. The mean of the income level is 1.94 with a standard deviation of 0.481, the mean of the family size is 9 with a standard deviation of 1.867, and the mean value of marital status is ~1 with a standard deviation of 0.50. Family size indicates a devastated and alarming health situation among Afghan refugees.

**TABLE 4:** Mean and SD of education level, income level, family size, and marital status

Socioeconomic factors	Mean ± SD
Education level	1.93 ± 0.250
Income level	1.94 ± 0.481
Family size	9.33 ± 1.867
Marital status	1.54 ± 0.500
Total	150

Abbreviation: SD, standard deviation.

### 3.2 Anthropometric assessment

#### 3.2.1 Body mass index

**FIGURE 2:**
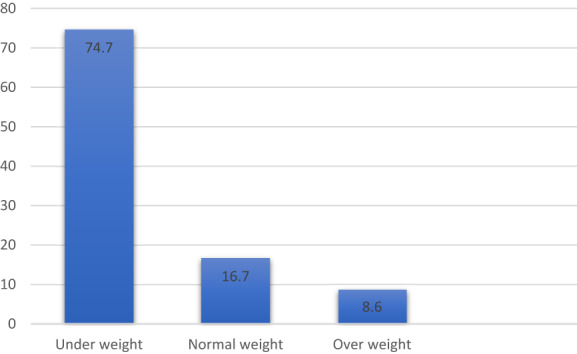
Bar graph showing BMI (body mass index) percentage and age based on categories

BMI is the most important factor in determining the nutritional health status of an individual and helps in indicating the risk of developing chronic conditions such as diabetes, hypertension, depression, and cancer (Khanna & Rehman, 2021). The weight and height of Afghan women based on BMI were calculated, and surprisingly the majority of the women were underweight; 52% of the participants belonged to the 15‐ to 20‐year age group, about 29.33% to the 21‐ to 25‐year age group, and 18.67% to the 26‐ to 30‐year age group. The height of most of these participants ranged from 5 ft to 5 ft 2 in. The weight results showed that 36.6% of the participants were in the range of 38–42 kg, and a few participants were 63–72 kg; 55 kg is the ideal weight, but only 4% of participants had this ideal weight. Figure 2 shows a bar graph with percentage of BMI and age based on categories.

The graph shows variations in BMI, and the highest percentage of underweight participants was 74.7%. Adolescents, aged 15–20 years, were underweight; 16.7% of participants had a healthy weight, and 8.6% were overweight. BMI ratios were consistent with other studies in adolescent Afghan refugees living in Peshawar, where stunting, thinness, and overweight and obesity were 35.3%, 4.4%, and 14.8%, respectively, as underweight ratio is also high in this research study (Saeedullah et al., 2021).

Table 5 shows the mean and standard deviation of age, weight, height, and BMI. The mean age of the sample is ~20–21 years with a standard deviation of 4.086, the mean weight is ~44.59 with a standard deviation of 7.930, the mean height of the participants is 60.49 cm with a standard deviation of 2.471, and the mean BMI of the participants is 18.797 with a standard deviation of 2.7499 (Al‐Attar et al., 2020).

**TABLE 5:** Mean and SD of age, height, weight, and BMI

Categories	Mean ± SD
Age	20.93 ± 4.086
Weight	44.59 ± 7.930
Height	0.49 ± 2.471
BMI	18.797 ± 2.7499

Abbreviations: BMI, body mass index; SD, standard deviation.

### 3.3 Biochemical assessment

**FIGURE 3:**
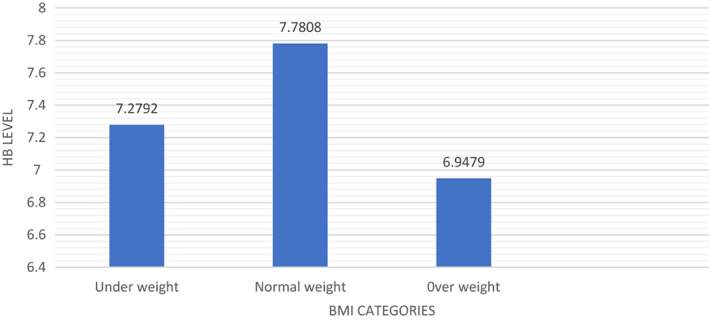
Bar graph showing mean of hemoglobin (Hb) level based on BMI (body mass index)

Hemoglobin concentration provides information on the degree of iron deficiency, making the prevalence of anemia an essential health indicator. Most of the underweight participants had a low Hb level (Khan, Khan, et al., 2018) as compared to normal‐weight participants. Technically, low hemoglobin is a result of iron imbalance in their bodies (Lynch et al., 2018). Overweight participants had the lowest level of hemoglobin due to the presence of additional fat in their bodies, and most of this fat is concentrated around the different organs. When oxygen enters the body, the process of this oxygen reaching the capillaries in the lungs is very slow, and the gas exchange system also automatically slows down, resulting in significant effects on RBC production. When the BMI increases, the serum levels of iron decrease, indicating a lower Hb level (Ausk & Ioannou, 2008). Different studies also confirm that iron deficiency is more common among overweight and obese adolescents and adults (Sumarmi et al., 2016). Figure 3 shows the mean of Hb level based on their BMI.

Figure 3 shows the variation in the Hb level based on their BMI categories, as normal‐weight participants have a mean Hb of ~7.7808 compared to overweight and underweight participants with 6.9479 and 7.2792, respectively. Hb level is high in overweight participants (Kumar, 2015).

### 3.4 Clinical assessment

Clinical assessment is one of the important steps in the assessment of nutritional status. It involves physical examination of hair, nails, skin color, and eye color. Different standards are used to examine these body parts. These factors are further discussed in detail.

#### 3.4.1 Skin color

The most important factor in examining the health status of individuals is their skin color. The options given in the questionnaire were healthy, dry, and pale yellowish. The majority of these participants had a pale‐yellowish skin, clearly indicating iron deficiency in blood, which is ultimately responsible for the low Hb level in the blood; data clearly show that even those who had a normal and healthy BMI had low Hb levels. It is well known that skin color is due to the RBC count, and if RBC count is low in the blood, then it ultimately results in a pale‐yellowish skin. One of the other reasons for their pale‐yellowish skin color is their unhealthy and insufficient diet pattern. Their diet was immensely affected by their environment, and also their living standards impacted greatly. Their diet was deficient in essential nutrients and minerals required for a healthy body. Most of these participants did not consume milk or other healthy foods, and even their water intake was very low; 57.33% had a pale‐yellowish skin, which is a clear indicator of low Hb and iron levels. About 26.67% of the participants had dry skin, and only 16% had a healthy skin.

#### 3.4.2 Hair, nails, and eye color

Hair is also one of the important factors in clinical examination, which indicates different types of deficiencies by its appearance. Research participants were asked if they had dry, dull, or thin hair or experienced extreme hair loss. About 38% of the participants had thin hair, and about 35.33% had extreme hair loss. About 13.33% of the participants had a dull and dry appearance, indicating that they also had protein and other essential nutrient deficiencies that are important for hair growth. The nails were analyzed physically, and about 54% of the participants had spoon‐shaped nails, meaning nails are slightly bent from the middle giving a spoon‐like appearance. About 26.67% of the participants had pink nail beds, and about 17.33% of the participants had smooth nails. Both pink nail beds and smooth nails had no transverse lines due to protein deficiency, and both types fall under the healthy category. Eye color was analyzed; the results indicated that about 88% had clear white eyes; about 8% had redness in their eyes, and about 4% had red edges. These data show variations in the eye color of the participants (Table 6).

**TABLE 6:** Mean and SD of hair, nail, eye, and skin

Clinical factors	Mean ± SD
Skin appearance	2.41 ± 0.753
Hair	2.95 ± 1.012
Nail	2.29 ± 0.863
Eye	1.16 ± 0.465
Total	150

Abbreviation: SD, standard deviation.

Table 6 shows that the mean and standard deviation of hair are higher, which is ~2.95 ± 1.012, and the mean and standard deviation of eyes are lower, which is ~1.16 ± 0.465.

### 3.5 Dietary assessment

This is the last part of the Anthropometric, Biochemical, Clinical and Diet (ABCD) nutritional assessment. Participants were asked about their diet history, daily diet routine, and dietary habits during this research study. Participants were asked about the type of diet, food groups, and major and minor meals they had. These Afghan refugees did not have minor meals or snacks. They only had major meals that were deficient in nutrients required for a healthy diet. Their appetite was measured based on the 24‐h dietary recall as it was a survey‐based study that was easy and could immediately measure their appetite.

#### 3.5.1 Dietary habits

Afghanistan is a landlocked country where people do not commonly consume seafood. They consume vegetables more than meat. As the poverty level is very high in their home country and also in Pakistan, all food groups are not available and affordable. The people usually eat bread loaf (*khamiri roti*) daily even without any side dish.

About 38.67% of the participants eat *khamiri roti* and herbal tea (*qahwa*) for breakfast, and about 10.67% eat *khamiri roti* with yoghurt. About 21.33% consume *qahwa* early morning, so they do not have breakfast but have lunch at about 12:00 noon; 48% of the participants eat *khamiri roti* with gravy for lunch, and about 10.67% skip their lunch. About 16% of the participants take rice with salad for lunch. The salad contains any of the seasonal vegetables like cucumber, onion, and tomatoes. If the prices of vegetables increase, then they skip the salad. Table 7 presents the dinner routine of the participants; about 48% eat *khamiri roti* and gravy for dinner, too. About 24% skip their dinner daily.

**TABLE 7:** Relationship between appetite and BMI using *χ* ^2^ test

Appetite type	Under weight	Normal weight	Over weight	Total
Normal	18	12	10	40
Percentage within the group	45%	30%	25%	100%
Suppress	90	13	3	106
Percentage within the group	84.9%	12.3%	2.8%	100%
Lack of interest	4	0	0	4
Percentage within the group	100%	0%	0%	100%
Total	112	25	13	150
Percentage within the group	74.7%	16.7%	8.7%	100%

Abbreviation: BMI, body mass index.

Participants’ dietary habits presented in Table 7 show that they eat *khamiri roti* more than anything else for breakfast, lunch, and dinner, and they drink *qahwa* more than water. They are addicted to this herbal tea that they consume it from early morning until night time. Their diets do not have all the food groups, and all the required nutrients are deficient in their diets. *Khamiri roti* contains a major portion of carbohydrates, some portion of proteins, and low amounts of fat. It is good for weight loss and diabetic patients. The bread is made from whole wheat flour, yeast, and milk, but only these are not sufficient to fulfill the nutrients required for good health.

Table 7 shows Pearson's *p‐*value of .00 with a *χ* ^2^ value of 29.539, indicating a strong relation between BMI category and the type of appetite as the majority of participants have a deficient diet and low quantities of food.

#### 3.5.2 Any food allergies

As these refugees are living in extreme poverty, a majority of them do not have any kind of food allergies from any specific food, but there were a few rare cases where some food allergies were observed; some adults, teenagers, and even a few children were allergic to gluten. These participants encountered affordability and accessibility problems. The condition of these participants was measurable and very critical. The inadequate income of these refugees, the large family sizes to be fed, and the basic needs to be met make it impossible to consume gluten‐free diets. About 95.33% of the participants did not have food allergies. About 4.67% had food allergies toward some specific foods.

#### 3.5.3 Water intake

Afghan refugees are very addicted to drinking *qahwa*. They drink water less than the daily requirement for a healthy life. They drink *qahwa* even early morning, and people of all age categories consume this tea. The data in Table 8 clearly show that about 86.67% of the participants, which is more than half of the targeted participants, drink only two to four glasses of water per day; this water consumption level is very less. Only 1.33% of the participants drink six to eight glasses of water per day, which is inadequate. About 86.67% consume only two to four glasses of water per day, and about 1.33% consume only six to eight glasses of water per day.

**TABLE 8:** Mean and SD of breakfast, lunch, dinner, water intake, and food allergies

Dietary assessment factors	Mean ± SD
Breakfast	2.15 ± 1.155
Lunch	1.89 ± 1.031
Dinner	2.31 ± 1.074
Water intake	1.10 ± 0.380
Any food allergies	1.95 ± 0.212
Total	150

Abbreviation: SD, standard deviation.

Table 8 shows that the mean and standard deviation of breakfast is higher among all other factors, which is ~2.15 ± 1.155, and the mean and standard deviation of water intake is the lowest among all, which is 1.10 ± 0.380.

### 3.6 Infection rate among participants

Some kinds of infections, such as UTIs (urinary tract infections) and skin infections, were experienced by the majority of the research participants. A majority of these participants in the age group from 15 to 30 years had UTI infections, but they were high in adolescent girls. Unhygienic conditions are the main reason for UTIs. Some participants had skin infections, like allergies on skin. About 56.67% of the participants had infectious diseases.

Table 9 shows that about 56.67% of the participants had UTIs, which is more than half of the participants. About 18% of the participants had no infections.

**TABLE 9:** Infectious disease ratio among participants

Infection rate	Frequency	Percentage
UTI	85	56.67
Other than UTIs	38	25.33
Nothing	27	18
Total	150	100

Abbreviation: UTI, urinary tract infection.

### 3.7 Other conditions related to health

A total of 67 participants (44.68%) had headache, and almost 66 participants (44%) had tiredness throughout the day. About two participants (1.33%) in each category had depression and diarrhea, and some did not experience any medical problems. Eleven participants (7.33%) experienced nausea whenever they consumed food or even when on empty stomach.

Above Table 10 presents the *p‐*value of .05 with the *χ* ^2^ value of 17.508, meaning that the aforementioned health conditions were significantly attributed to BMI; 87.9% of underweight participants experienced tiredness, and 66.2% of underweight participants had extreme headache; tiredness is also a direct indicator of low Hb ratio.

**TABLE 10:** *χ* ^2^ Test showing relation between BMI and health‐related conditions

Health condition	Under weight	Normal weight	Over weight	Total
Depression	3	2	0	5
Percentage within their health condition	60%	40%	0%	100%
Headache	43	13	9	65
Percentage within their health condition	66.2%	20%	13.8%	100%
Diarrhea/dysphagia	5	4	2	11
Percentage within their health condition	45.5%	36.4%	18.2%	100%
Feeling tired	58	6	2	66
Percentage within their health condition	87.9%	9.1%	3%	100%
Nausea	1	0	0	1
Percentage within their health condition	100%	0%	0%	100%
Nothing	2	0	0	0
Percentage within their health condition	100%	0%	0%	0%
Total	112	25	13	150
Percentage within their health condition	74.7%	16.7%	8.7%	100%

Abbreviation: BMI, body mass index.

### 3.8 Any history of family disease

**FIGURE 4:**
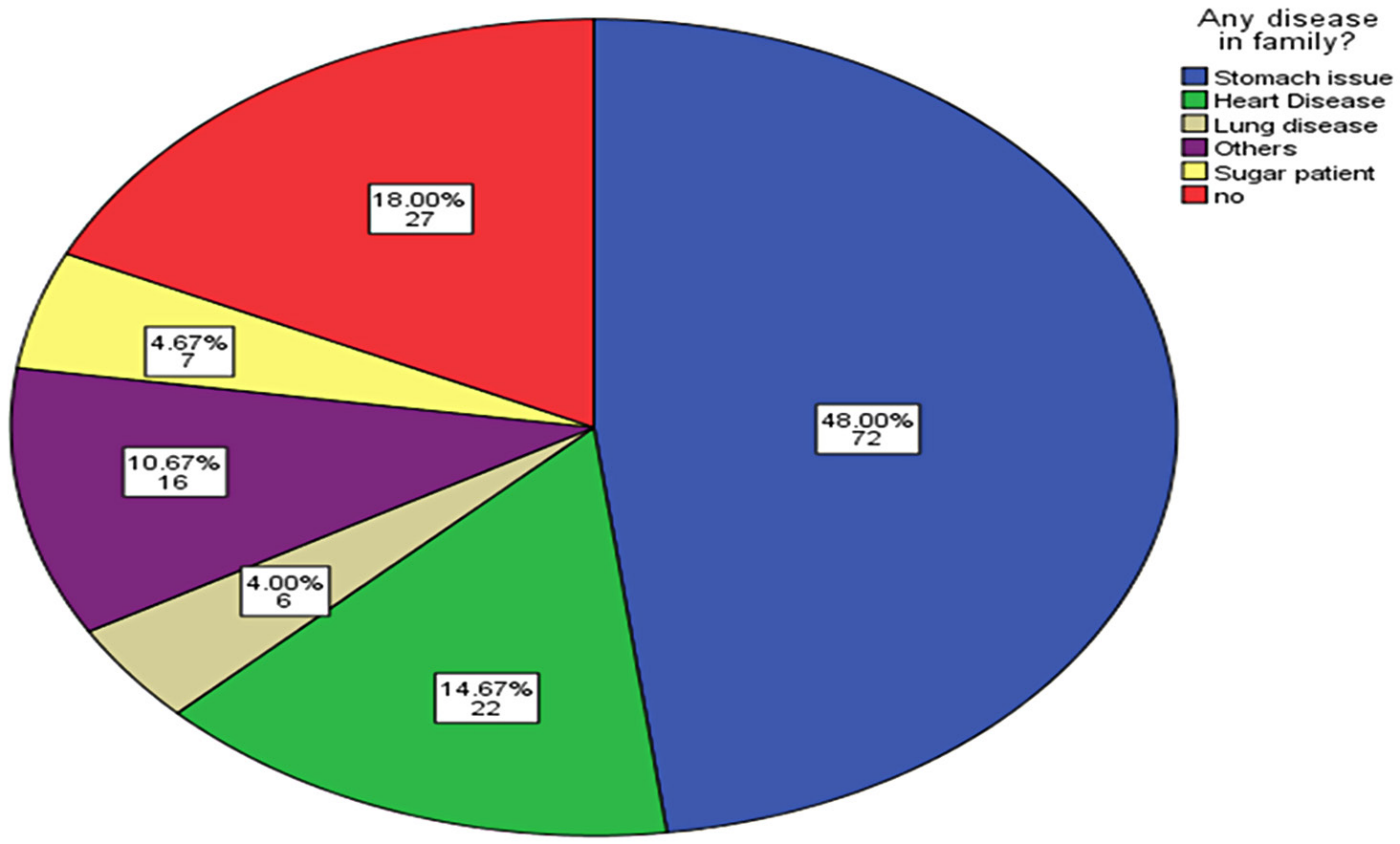
Pie chart showing a list of disease ratios among families

Figure 4 shows the number of diseases in the families of the participants and the variations among them. A majority of the participants had stomach‐related issues. About 27 participants had no specific disease in their family history. About 48% of the participants experienced stomach issues, which were also observed in their family. About 4.67% of the participants had diabetic patients in their families. Almost 10.67% of the participants had some other health‐related issues in their families, like fever, any joint pain, and any kind of surgery. About 4% had lung disease in their family history, and about 14.67% had heart‐related issues in their family. Figure 4 shows a list of disease ratios among families.

### 3.9 Allergy scoring

Because only a few participants experienced allergy during this study, we do not discuss it here.

### 3.10 Recommendations

Besides anthropometric measurement, other methods of nutritional assessment like 7‐day recall method and 24‐h recall method were used. But 24‐h recall method is much better than 7‐day recall method. More details of food intake should be recorded using food frequency questionnaires.

Further studies involving biochemical and clinical analyses such as hemoglobin level, fraction of plasma protein, blood calcium level, and lipid profile of mothers as well as in newborns are required to access their immunity and health data.

Longitudinal studies should be conducted by registering women's age of puberty for better results.

## 4 CONCLUSION

This study aimed to highlight the nutritional health status of Afghan refugees living in Punjab, Pakistan, because the nutritional health of women has a strong impact on overall family health status. A large number of research studies related to the health of the Pakistani population are conducted every year in different universities in different cities of Pakistan. But no study has highlighted this community that is present in Pakistan since 1980. Their income level, education, dietary habits, and lack of interest in food have an impact on their BMI, and their physical appearance also clearly indicates their Hb level. Their extremely poor Hb level is a direct predictor of RBC count in the blood. This indicates the anemic condition, especially iron deficiency, among young Afghan refugee women. This study will help future researchers find out better ways of analysis and treatment plan for such communities who are highly prone to nutritional deficiencies and lack a healthy life. Further studies should highlight the main health‐related issues of these refugee women due to limited supply of resources.

## CONFLICT OF INTEREST

Authors declare that they have no conflicts of interest.

## FUNDING INFORMATION

The authors declare that no funds, grants, or other support were received during the preparation of this manuscript.

## CONSENT TO PARTICIPATE

All the coauthors were involved in this study.

## CONSENT FOR PUBLICATION

All authors provided their consent for publication of this manuscript.

## Data Availability

Even though adequate data have been given, all authors declare that if more data are required, then the data will be provided based on request.
